# Understanding the healthcare provider role on post abortion contraception adoption in India using National Family Health Survey-5

**DOI:** 10.1186/s12978-023-01667-z

**Published:** 2023-08-23

**Authors:** Anjali Bansal, Arpana Kullu, Priyanka Dixit

**Affiliations:** 1 Research Scholar, International Institute for Population Sciences, Govandi Station Road, Deonar, 400088 Mumbai India; 2 Research Scholar, Tata Institute of Social Sciences, V. N. Purav Marg, Deonar, 400088 Mumbai India; 3 Assistant Professor, School of Health Systems Studies (SHSS), Tata Institute of Social Sciences, V. N. Purav Marg, Deonar, 400088 Mumbai India

**Keywords:** Post-abortion contraception use, Health workers, Reproductive Calendar Data, National Family Health Survey, India

## Abstract

**Background:**

Post abortion contraceptive use is an important area in provisioning of services associated with child birth planning. This study examines the factors related to the type and timing of initiation of contraception adoption among women who had undergone induced abortion. Study also tries to identify the role of personnel who provided the abortion service on decision of family planning adoption using complementary log–log model in India.

**Methodology:**

The study uses the secondary data from the fifth round of the National Family Health Survey conducted during 2019–21. For, the analysis, we have used five-year women’s reproductive calendar to extract information on contraceptive use, post last induced abortion among women. We used complementary log–log regression models, to estimate relative risk ratios and its 95% Confidence intervals (CI).

**Results:**

According to NFHS-5, out of all the last pregnancies (2,55,549), about three percent resulted in abortion. Most of the abortion occurred in private facilities (55%), with the help of health professionals (71%). From the women’s reproductive calendar, it was found that around 40% of the women adopted modern methods of contraception, with maximum adopting spacing method (33%), and only handful adopted permanent method (7%). It was also found that the likelihood of early adoption of permanent method increased to two times when the abortion is done by health professional compared to others [95% CI (1.25–3.30)].

**Conclusion:**

This emphasises a need for quality counselling related to timing and types of family planning as an essential part of the family planning program ensuring client centric approach suited to their needs and contexts that helps in alleviating any apprehensions associated with adverse effects of modern contraceptive methods.

**Supplementary Information:**

The online version contains supplementary material available at 10.1186/s12978-023-01667-z.

## Introduction

Abortion and contraception are two significant reproductive healthcare services which are associated with limiting or spacing of child bearing for any couple. Both these services are interlinked as the unmet need for contraception is documented as a major proximate determinant of the untimed pregnancies and induced abortions [1–4]. Most induced abortion follow from pregnancies which were unwanted or mistimed as they were conceived earlier than desired, due to no use or failure of contraception [5, 6]. Worldwide of every four pregnancies, one pregnancy is identified as being unintended as per reports by the World Health Organisation (WHO) [7]. Recent estimates around the world shows that the proportion of unintended pregnancies ending in abortion is around 68% [8], which illustrates the need to strengthen family planning services to avoid these unplanned pregnancies. Using data from health facility survey, NGO clinic, National medication abortion drug sales and distribution, a study estimated about 15.6 million abortion in India in 2015. This study also estimated that the abortion rate was 47.0 per 1000 women aged 15–49 years [9].

Another significant aspect associated with abortion is the post abortion care (PAC), which is recognised as an approach to improve women’s sexual as well as reproductive health, by ensuring that there are family planning services available to avoid unplanned pregnancies in future. WHO recommends provision of contraceptive methods and appropriate counselling for all those women who wish to prevent unintended pregnancies and thereby avoiding going through abortion. The integration of family planning (FP) counselling and method provision into abortion services is an essential part of PAC [10]. In this regards the study becomes essential to study the contraceptive practices and preferences among currently married women who undergo abortion. Evidence shows that PAC services only focuses on the emergency treatment and neglected the family planning counselling [11, 12]. Studies in India based on Demographic Health Survey (DHS) also show very low contraception use post abortion [13, 14].

Among the users of various family planning methods, mostly women adopted short acting modern methods followed by traditional methods in India [14]. Moreover, sex composition of living child and socio-economic variables influence contraception adoption as well [12, 15, 16]. In India, post abortion timing and choice of contraception adoption depends upon choice of provider including place and person who performed abortion and procedure adopted for abortion [17, 18]. Moreover, out of all these components, person who performed abortion play very crucial role. As it can be understood that when abortion services are provided by a skilled healthcare provider they are more likely to provide adequate and appropriate information about the various options available and which may be more aptly suitable to the needs of the couple.

Therefore, the main aim of the study is to understand the linkages between choice of provider for abortion service and post abortion contraceptive use and its associated constructs amongst Indian women. We also determine the timing of adoption of various type of family planning methods using complementary log–log model. This would also help healthcare providers to include appropriate strategies and clinical protocol in provisioning post abortion care and family planning services to ensure that people centric approaches are adopted to encourage positive health behaviours.

Further, the publication of the latest data from the fifth round of National Family Health Survey is timely wherein the information about reason of abortion, procedure used for abortion, and person who performed abortion is collected for the first time. Using this information, we hypothesized that women who undergo abortion by a skilled health professional tend to adopt a modern method early as they may receive counselling about the contraceptive method under PAC compared to those who do not undergo abortion procedure through a skilled healthcare provider.

## Materials and methods

### Data source

The data used for the analysis is the fifth round of the NFHS (2019–2021). It is a nationally representative cross-sectional survey which includes representative sample of the households throughout India. The survey provides state, national and district level estimates of demographic, health as well as socio-economic status and program dimensions, which are critical for implementing the desired changes in demographic and health parameters. Stratified, two-stage sampling is mostly used in all DHS surveys to obtain a representative sample of households. Probability proportional to size (PPS) was used to select the households from all states and Union Territories. Within each rural stratum, villages were selected from the sampling frame with PPS. In urban areas, Census Enumeration block (CEB) were selected based on data from the census of India (the detailed sampling method is available here [19]). The survey for the first time in NFHS-4 (2015–2016) provided data on key indicators associated with the demographic and health parameter at district level which continued in NFHS-5.

### Analytical sample

The NFHS fifth round collected data from 512,408 currently married women out of them 7489 had undergone induced abortions in last five years. These were selected for analysis in the current study, wherein we have only selected the last abortion occurred in the five years preceding the date of interview.

### Study variables

#### Outcome variable

In this study, we have used the retrospective reproductive history of women to assess the time to adopt modern contraceptive methods after the last abortion. NFHS-V uses the concept of a contraceptive calendar, which consists of usually 60–70 months history of a women’s reproductive behaviour, including her timing of pregnancy, contraceptive behaviour, and pregnancy termination (abortion, miscarriage, and stillbirth). The contraceptive calendar contains information about all the reproductive events for the last five years prior to the date of the interview. The information presented in the calendar was in string format. The position of last termination (inducted abortion) was extracted from the calendar column. The outcome variable of interest was the time to adopt the different contraceptive methods after the last termination. The modern method included sterilization, injectables, intrauterine devices, pills, condoms, diaphragm, foam/jelly, and other modern methods.

#### Explanatory variables

In the study various explanatory variables were included. The household level variables included Place of residence (Urban, Rural), Region (North, Central, East, North-East, West, and South), Religion (Hindu, Non-Hindu), Caste (Scheduled Caste (SC)/Scheduled Tribes (ST), Other Backward Caste (OBC), Others (other then SC/ST and OBC), Household Wealth Quintile (Poorest, Poorer, Middle, Richer, and Richest).

The individual-level factors included Age of women in completed years (15–29, 30–39, 40 +), Educational Status of women (No education, up to Primary, above primary and up to Secondary and Higher and above), Parity of women (Less than two children, More than 2 children), Sex Composition (No child, Only son, Only daughter, Both son and daughter).

In NFHS-5, some new questions were added to obtain more information about abortion which were relevant to understand why and where women have undergone abortion. So in the analysis we have also included information about, Age of the woman during abortion in completed years (less than 20, 21–24, 25–29, 30 +), Place of abortion^1^ (Public facility, Private facility, Home), Person who performed abortion^2^ (Health professional, Self, others), the procedure used for abortion (Surgical, non-surgical), and reason for abortion (Unplanned pregnancy, Medical Issues, Others).

### Statistical analysis

In this study, we calculated the time to adopt the modern method of contraception after the last abortion. Given the discrete nature of data and time which was captured in months between abortion and contraception method adoption, we used complementary log–log regression models, to estimate relative risk ratios and its 95% Confidence intervals (CI). The complementary log–log model is a discrete-time survival analysis similar to the continuous time Cox proportional hazards model [20]. We have run three models to determine the time to adopt a specific contraception method. In the First model, outcome variable was spacing method vs. no method (no method and traditional method), in second model outcome variable was permanent method vs. no method and for third model we included any modern method vs no method. Data analysis was performed using Stata software version 17.0. All the results were reported at a 95% confidence interval. Since the NFHS includes a complex sampling design and has an effect on the confidence intervals, so we considered the complex survey design of NFHS within svyset and svy procedures in Stata [21].

## Results

### Characteristics of the sample

**Fig. 1:**
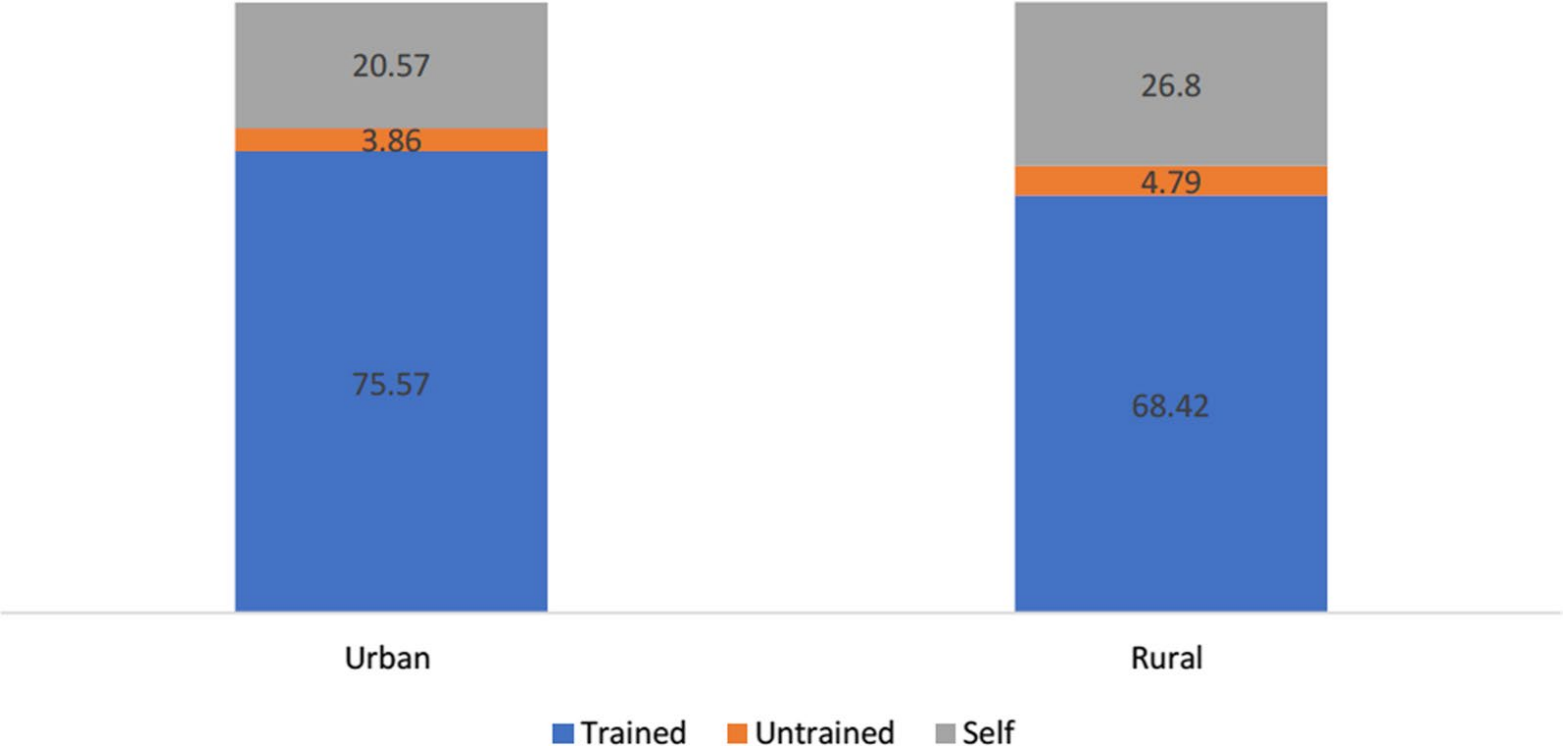
Percentage distribution of person who performed abortion by place of residence NFHS-5, 2019-21

About 24.4% women who have undergone abortion belong to the Central region and about 23.7% belong to the Eastern regions of India. Nearly two-thirds of the women (62.7%) undergoing abortion resided in rural parts of India. Higher proportion of women belong to the upper wealth quintile underwent abortions (43%). Around two-thirds of the women (60%) less than 29 years of age undergo abortion, with the mean age of women of abortion as 27.8 years [95% CI (27.6–27.9)]. Nearly 86% of the respondents had undergone an abortion in the first trimester of their pregnancy. Nearly two-thirds of the women (62%) were found to get abortions done after two parity in the last five years. More than half of the women (55.2%) preferred private health facility for abortions and mostly did their abortions by health professionals (71%) through the non-surgical procedure, medicines (67.4%), and other methods (4.6%). Nearly half of the women (43%) had their abortion done because of an unplanned pregnancy (Table 1). Our findings indicate that there is a notable difference in abortion practices between urban and rural areas. Figure 1 presents the percentage distribution of person who performed the abortion by place of residence, and it was found that 76% of women in urban areas sought abortion services from trained professionals such as doctors, nurses, and ayush practitioners. In contrast, in rural areas, the percentage of women accessing abortion from trained professionals was 68%, while a significant 27% of women in rural areas opted to perform abortions on their own without any professional assistance.

**Table 1:** Background characteristics of currently married women aged 15–49 who had induced abortion in the last five years in India, NFHS-5, 2019–21

Place of residence	Unweighted frequency	Percent
Urban	3127	37.3
Rural	5260	62.7
Region		
North	953	11.4
Central	2044	24.4
East	1983	23.7
North East	484	5.8
West	1189	14.2
South	1733	20.7
Religion		
Hindu	7081	84.4
Non-Hindu	1306	15.6
Caste		
SC/ST	2416	28.8
OBC	3564	42.5
Others	2407	28.7
Wealth quintile		
Poorest	1134	13.5
Poorer	1594	19.0
Middle	1774	21.2
Richer	1955	23.3
Richest	1930	23.0
Age of women (in completed years)		
15–29	4886	58.3
30–39	3124	37.3
40 +	376	4.5
Educational status of women		
No education	1102	13.1
Primary	964	11.5
Secondary	4670	55.7
Higher	1650	19.7
Parity of women		
Less than 2 children	3188	38.0
More than 2 children	5198	62.0
Sex composition		
No child	677	8.1
Only son	2518	30.0
Only daughter	1839	21.9
Both	3353	40.0
Ideal number of children in relation to actual living children		
Ideal = Actual	3333	44.6
Ideal is more than actual	2993	37.4
Ideal is less than actual	1370	18.0
Mean age at abortion (in completed years)	27.8, 95% CI (27.6–27.9)	
Timing of abortion		
First trimester	6575	85.7
Second trimester	1121	14.3
Place of abortion		
Public facility	1710	20.4
Private facility	4632	55.2
Home	2044	24.4
Person who performed abortion		
Health Professional	5936	70.8
Others/self	2450	29.2
Method used for abortion		
Surgical	2346	28.0
Non-Surgical	6040	72.0
Reason for abortion		
Unplanned pregnancy	4434	52.8
Medical issues	2583	30.8
Other*	1369	16.3
Total	8,386	100.0

^*^contraception failure, economic reasons, husband/mother-in-law don’t want, other

### Post-abortion contraceptive adoption

**Fig. 2:**
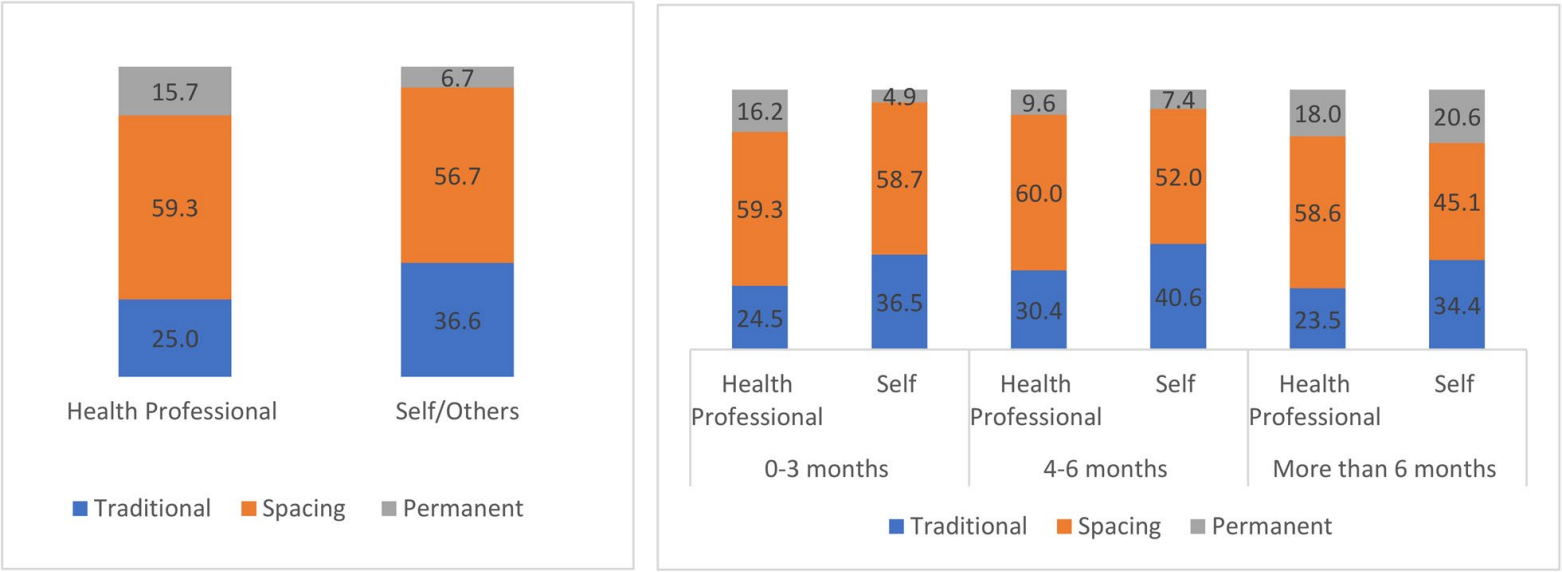
Percentage distribution of post abortion, time to adopt specific type of contraception adoption among women according to person who performed abortion in India, NFHS-5, 2019–21

The magnitude of contraceptive adoption among women who have had undergone an abortion was overall relatively low compared to women who did not undergo abortion in India (Additional file 1: S1). Post abortion 43.2% women prefer not to use any method, 40.4% chose modern method and 16.5% chose traditional spacing method. In the first three months post-abortion, two third of the women adopted the spacing method followed by the traditional method (28.7%). Only a handful of women adopted a permanent method of contraception in the first three months, while around 19% adopted female sterilization after six months post-last abortion (Additional file 2: S2). In case of women, wherein abortion was performed by health professionals, around 16% adopted a permanent method of contraception compared to only seven percent of women who adopted a permanent method if health professionals were not involved (Fig. 2). It was found that 16% of the women adopted female sterilization within three months post-abortion if their abortion was conducted by a health professional (Fig. 2).

### Factors associated with the post abortion, time to adopt modern contraceptive methods among currently married women

The bivariate analysis of the type of contraception method adopted post-abortion was presented in Table 2. Of 7696 women who underwent an abortion in the last five years, 60% of the women haven’t adopted any modern method of contraception, around one-third of the women adopted spacing method post abortion, and only a handful (7.2%) of the women used a permanent contraceptive method. It was found that more urban women were adopting any spacing method (36.6%) compared to rural (31%) women. About 38% of the women who belong to other caste were found to adopt spacing method. Compared to other levels of educated women, primary educated women were found to use more spacing  (36.5%) and permanent method (10.7%).

**Table 2:** Percentage distribution of type of contraception method adopted post abortion among currently married women in the last five years by explanatory variables in India, NFHS-5, 2019–21

Background variables	No method	Spacing method	Permanent method
Place of residence			
Urban	55.9	36.6	7.4
Rural	61.9	31.1	7.0
Region			
North	57.3	37.8	5.0
Central	53.7	40.7	5.6
East	54.3	37.2	8.5
North East	53.6	45.2	1.2
West	55.8	33.8	10.4
South	78.4	13.5	8.2
Caste			
SC/ST	59.0	32.4	8.7
OBC	63.5	30.5	6.0
Others	54.6	38.0	7.4
Religion			
Hindu	59.6	32.9	7.6
Non-Hindu	60.1	34.8	5.1
Age of women (in completed years)			
15–29	64.0	31.3	4.8
30–39	52.3	36.9	10.7
40 +	64.4	26.6	9.0
Educational status			
No education	61.9	28.0	10.1
Primary	51.1	36.5	12.4
Secondary	59.9	33.4	6.7
Higher	62.3	34.1	3.6
Child sex composition			
No child	81.3	18.7	0.0
Only son	57.9	36.6	5.5
Only daughter	64.3	32.1	3.7
Both	54.1	34.2	11.8
Parity of women			
Less than 2 children	66.6	32.5	1.0
More than 2 children	55.4	33.6	11.0
Ideal number of children in relation to actual living children			
Ideal = actual	51.7	37.7	10.7
Ideal is more than actual	70.1	28.4	1.5
Ideal is less than actual	57.7	32.0	10.3
Age at abortion (in completed years)			
Less than 20	74.0	25.0	1.0
21–24	66.6	29.2	4.3
25–29	56.4	35.2	8.4
30 +	55.2	35.6	9.2
Timing of abortion			
First trimester	57.6	35.1	7.3
Second trimester	72.1	21.5	6.4
Place of abortion			
Public facility	56.9	32.9	10.2
Private facility	61.0	31.5	7.5
Home	58.9	37.3	3.8
Person performed abortion			
Health professional	60.3	31.4	8.3
Others/self	58.1	37.4	4.4
Method used for abortion			
Surgical	57.4	32.9	9.8
Non-surgical	60.5	33.3	6.2
Reason for abortion			
Unplanned pregnancy	51.5	40.2	8.3
Medical issues	74.4	20.5	5.1
Others	58.2	34.2	7.6
Total	59.6	33.2	7.2

Among women who underwent abortion in public health facilities, those who received abortion services from health workers and underwent surgical abortion had a higher percentage of adopting sterilization, compared to their counterparts.

The multivariable complementary log–log model was presented in Table 3. A total of three models were run to show the type of contraceptive method women adopted early post-abortion. For model 1, the outcome variable was time to adopt the spacing method. All the explanatory variables were found to be significantly associated with the adoption of the spacing method except caste, religion, the timing of abortion, the person who performed the abortion, and the method used for abortion.

**Table 3:** Multivariable Logistic regression for adoption of female sterilization post abortion in last five years by selected covariates in India , NFHS, 2019–21

	Model 1	Model 2	Model 3
Background variables	Spacing method Vs No method	Permanent method Vs No method	Any Modern Method Vs No method
	AOR (95% CI)		
Place of residence			
Urban	1.00	1.00	1.00
Rural	0.83**(0.70–0.98)	0.81 (0.59–1.13)	0.84**(0.72–0.99)
Region			
Northern	1.00	1.00	1.00
Central	1.03 (0.83–1.29)	1.07 (0.67–1.72)	1.03 (0.84–1.28)
Eastern	0.97 (0.77–1.22)	2.19***(1.39–3.46)	1.09 (0.88–1.35)
North-Eastern	1.12 (0.88–1.42)	0.22***(0.10–0.50)	1.01 (0.81–1.27)
Western	0.72**(0.54–0.96)	2.28***(1.29–4.05)	0.88 (0.67–1.16)
Southern	0.43***(0.33–0.57)	4.21***(2.54–6.98)	0.70***(0.54–0.90)
Caste			
SC/ST	1.00	1.00	1.00
OBC	1.08 (0.91–1.28)	0.75 (0.53–1.06)	1.01 (0.86–1.19)
Others	1.17 (0.97–1.41)	1.34 (0.87–2.06)	1.18*(0.99–1.42)
Religion			
Hindu	1.00	1.00	1.00
Non-Hindu	1.06 (0.86–1.32)	0.58*(0.33–1.03)	0.95 (0.76–1.19)
Age of women (in completed years)			
15–29	1.00	1.00	1.00
30–39	0.64***(0.50–0.81)	0.97 (0.64–1.47)	0.70***(0.56–0.88)
40 +	0.47***(0.32–0.7)	0.79 (0.34–1.82)	0.53***(0.36–0.77)
Educational status			
No education	1.00	1.00	1.00
Primary	1.25 (0.96–1.64)	1.12 (0.75–1.66)	1.24*(0.96–1.59)
Secondary	1.27**(1.02–1.59)	0.64**(0.46–0.90)	1.13 (0.92–1.39)
Higher	1.47***(1.12–1.92)	0.42***(0.23–0.79)	1.19 (0.93–1.53)
Child sex composition			
No child	1.00		1.00
Only son	1.16 (0.82–1.64)	1.00	1.25 (0.89–1.77)
Only daughter	1.45**(1.02–2.05)	0.86 (0.61–1.21)	1.49**(1.05–2.11)
Both	1.29 (0.88–1.87)	0.71 (0.42–1.21)	1.42*(0.98–2.08)
Parity of women			
Less than 2 children	1.00	1.00	1.00
More than 2 children	0.62***(0.47–0.82)	5.08***(2.69–9.61)	0.79*(0.61–1.02)
Ideal number of children in relation to actual living children			
Ideal = actual	1.00	1.00	1.00
Ideal is more than actual	0.80 (0.61–1.05)	0.72 (0.46–1.15)	0.80*(0.62–1.02)
Ideal is less than actual	0.86*(0.71–1.03)	0.79 (0.57–1.10)	0.84*(0.71 1.01)
Age at abortion			
Less than 20	1.00	1.00	1.00
21–24	0.94 (0.70–1.26)	2.77 (0.63–12.22)	0.99 (0.73–1.33)
25–29	1.15 (0.87–1.52)	3.94*(0.88–17.66)	1.24 (0.94–1.64)
30 +	1.57**(1.09–2.26)	4.03*(0.82–19.84)	1.56**(1.09–2.24)
Timing of abortion			
First trimester	1.00	1.00	1.00
Second trimester	0.90 (0.70–1.16)	0.81 (0.53–1.25)	0.90 (0.72–1.12)
Place of abortion			
Home	1.00	1.00	1.00
Public facility	0.73*(0.53–1.01)	1.33 (0.77–2.32)	0.82 (0.60–1.12)
Private facility	0.79 (0.60–1.06)	0.86 (0.53–1.40)	0.79 (0.59–1.05)
Person performed abortion			
Others/self	1.00	1.00	1.00
Health professional	1.14 (0.86–1.50)	2.03***(1.25–3.30)	1.24 (0.94–1.64)
Procedure used for abortion			
Surgical	1.00	1.00	1.00
Non-Surgical	1.00 (0.83–1.19)	0.59***(0.41–0.85)	0.90 (0.75–1.07)
Reason for abortion			
Unplanned pregnancy	1.00	1.00	1.00
Medical issues	0.72**(0.58–0.88)	0.87 (0.56–1.32)	0.71**(0.57–0.87)
Others	1.05(0.87–1.25)	1.06 (0.71–1.62)	1.05(0.88–1.26)

^*^p < 0.10, **p < 0.05, ***p < 0.00

The likelihood of time to adopt spacing method varies by educational status; highly educated women were more likely to early adopt spacing method than women with no education [AOR-1.47, 95% CI (1.12–1.92)]. Also, the sex composition of living children has a significant impact on time to adopt early contraception in India; women with only a daughter were more likely to early initiate spacing method than women with no children [AOR-1.45, 95% CI (1.02–2.05)]. Women with more than two parity were less likely to initiate a spacing method in India [AOR-0.62, 95% CI (0.47–0.82)]. It was found that if women undergo abortion after their 30 s, the likelihood of early initiation of spacing method increased to 1.57 odd times [95% CI (1.09–2.26)]. Also, if the place of abortion is a public facility, the odds of adopting spacing decreased to 27% compared to women who undergo abortion in their homes.

In Model 2, the outcome variable was time to adopt the permanent method of contraception. For sterilization, the adoption was high in the southern region of India compared to the Northern region [AOR-4.21, 95% CI (2.54–6.98)]. Among the secondary educated women, the odds of early adoption were lower compared to non-educated women [AOR-0.64, 95% CI (0.46–0.90)]. It was found that for women with a Parity of more than two, the odds of early adoption of the permanent method increased to five manifolds [AOR-5.08, 95% CI (2.69–9.61)]. In this study, we hypothesized that women who undergo abortion in the health facility and from a health professional were found to be using the permanent method of contraceptives as health professionals might advise women with higher Parity to undergo sterilization rather than multiple abortions if they do not want the child anymore. Along similar lines, we found that for women who undergo abortion by a health professional, the early adoption of the permanent method increased to two-manifolds [AOR-2.03, 95% CI (1.25–3.30)]. Also, women who undergo a non-surgical method of abortion were found to be less likely to adopt the early permanent method [AOR-0.59, 95% CI (0.41–0.85)].

In model 3, the dependent variable was the time to adopt any modern method of contraception. It was found that older women (40 +) were less likely to adopt the early modern method [AOR-0.53, 95% CI (0.36–0.77)] compared to younger women. Also, women who undergo an abortion at later ages were found to adopt any modern method earlier than those who undergo an abortion at their early ages [AOR-1.56, 95% CI (1.09–2.24].

## Discussion

This study investigates the factors affecting post abortion, contraception use, based on the health personnel who provided the abortion service among women using complementary log–log model in India. Additionally, the study also examines the time of uptake of contraception by women, who had undergone induced abortions in the last five years from different socio-demographic groups to provide insights into the beneficiaries social profile and the factors influencing the same. According to PAC service, all women should be counselled about the different contraception methods [22]. Despite this provision, only around 40% of the women adopted modern method of contraception, with maximum adopting spacing method (33%), and only handful adopted permanent method of sterilization (7%). The complementary log–log analysis found that the odds of early adoption of permanent method increased two manifolds when abortion was done by a health professional compared to those done their abortion on their own/others 95% CI (1.25- 3.30)). Similar to our findings, studies have found that women resume sexual activities soon after abortion, and majority did not adopt any method [23–25]. These findings can be explained as government only focused on permanent method of contraception through their National Family Health welfare programme [15, 26], and not motivate women to adopt any spacing method despite their parity, which demonstrates that the government’s efforts to provide temporary method were futile [27, 28]. Also, the incentives given to women in female sterilization could be a motivating factor behind the uptake of the contraception method. Earlier studies in India have hinted that the women adopt sterilization due to the huge incentives involved in it [29, 30]. Also, in our study we found that women were more likely to adopt sterilization if they had received incentives for sterilization (Additional file 3: S3), which can later result in regret. A recent study in India had found that the amount of compensation is the major factor in sterilization regret [31]. Though women adopting sterilisation post abortion may not be a high proportion but it may present as a viable option for those undergoing recurrent abortions for unplanned pregnancies; which in itself needs to be explored further through research conducted in different contexts.

The analysis also indicated that the type of procedure of abortion plays an important role in determining the initiation of modern spacing method, where women who have gone through non-surgical method of abortion was 40% less likely to early initiate modern permanent method (95% CI – 0.41 to 0.85). Similar to our findings, a study in Bihar and Jharkhand, India have found that women who have undergone non-surgical abortion were less likely to adopt any modern method post abortion [17]. While contrary, a more recent study found that there are no differences in the contraception adoption by the procedure of abortion [25].

Results from this study also indicated that 53% of the women opting for abortion because of the unplanned pregnancy. Of them 52% of them still did not initiate use of any modern method of contraception. It is intriguing to note that women who do not use any method post abortion and did not wish to have last pregnancy might result in another unplanned pregnancies and subsequent abortion [32]. Around 17% of the women used traditional method which puts women at the risk of repeated abortions [14]. This findings indicate that there is a need to promote behavioural change among women to adopt an effective method post abortion to avert recurrent abortion which may have a negative impact on both maternal and child health [18, 33].

Study based on NFHS-4 shows that if women accessed abortion services in private clinic they were more likely to choose permanent method or IUCDs. The study also emphasised that there was a lack of choices and low uptake of contraceptives along with the likely dominance of permanent method. Thus helping to reiterate the findings of our study wherein similar preference is seen for permanent methods when the abortion was done by a health professional.

In this regard, health personnel’s play an important role in advocating positive health behaviours by counselling on the basket of choices available to both women and men. When women are reaching any health professional it means that they are in close proximity to the health system and thereby can be motivated with appropriate counselling to avert future unintended pregnancy. Several studies have shown that effective counselling and appropriate selection of client based on their contraceptive needs for either spacing methods or permanent methods can significantly reduce unintended pregnancies [34, 35]. Additionally studies have also noted that quality of counselling ensuring interpersonal communication, adequate time, frequency of counselling and number of methods during counselling need to be further investigated to ensure greater impact [34]. Study reveals that a good interpersonal counselling by the health providers has positive impact by way of alleviating fear about side effects of contraception in the minds of the beneficiaries [36]. This indicates an essential requisite for family welfare program and policy from the perspective of skilling health providers in providing counselling along with the knowledge about various contraceptive measure available. The study implies that health programs and policies need to promote spacing methods along with permanent method as younger women also under the  influence of health professionals adopt female sterilization method of contraception which later can result in regret [29]. Therefore, skilled counsellors for family planning can be seen as an essential part of the family planning program [36, 37]. Additionally, the study also highlights the need to adopt strategies for better uptake of contraceptive measures and to address untimed pregnancies. A recent study in China has demonstrated effectiveness of pre-abortion counselling and follow up of women after abortion [38]. The study emphasises that a reliable advice on contraception played an important role in contraceptive use. Pre-abortion counselling can also form an essential part of clinical protocol which prepares women to choose from various contraceptive measure to prevent untimed pregnancies.

The findings of this study needs to be read along with its limitations as well. Since the study uses the NFHS dataset to understand the post abortion contraceptive uptake based on the health personnel’s who conducted the abortion in last five years there are chances of recall error in reporting their contraceptive use as well. Additionally, our analysis was limited by the small sample size, making it challenging to examine the time to adopt contraception by the type of provider. Specifically, there were fewer than 500 cases of abortion conducted by untrained professionals, which impacted the statistical robustness of our findings in this particular category.

### Supplementary Information

**Additional file 1: S1.** Percentage distribution of contraception use, permanent, spacing, and traditional method among currently married women according to status of abortion in last five years in India, NFHS-5, 2019-21.

**Additional file 2: S2.** Percentage distribution of time to adopt a contraception method, permanent, spacing, and traditional method in last five years by type of contraception among women who undergo abortion in India, NFHS-5, 2019-21.

**Additional file 3: Table S1.** Adjusted Logistic regression for adoption of female sterilization post abortion in last five years by selected covariates in India, NFHS, 2019-21.

## Data Availability

The datasets analysed during the current study are from National Family Health Survey (NFHS) for India. The data is freely available from the DHS website, The DHS Program, India: Standard DHS, 2019–21 Dataset.

## Footnotes

1 Public Facility included, Government hospitals, AYUSH, Government dispensaries, UHC,UHP, UFWC, CHC, Rural Hospital, PHC, Sub-center, mobile clinics. Private facility included, Private Hospital, Clinics, Dispensaries, NGO.

2 Health Professionals included, Doctor, Nurse, ANM, AYUSH. Others included, Dai, Family, friends, and others.
