# Correction: Understanding the healthcare provider role on post abortion contraception adoption in India using National Family Health Survey-5

**DOI:** 10.1186/s12978-023-01683-z

**Published:** 2023-10-10

**Authors:** Anjali Bansal, Arpana Kullu, Priyanka Dixit

**Affiliations:** 1 International Institute for Population Sciences, Govandi Station Road, Deonar, 400088 Mumbai India; 2 Tata Institute of Social Sciences, V. N. Purav Marg, Deonar, 400088 Mumbai India; 3 School of Health Systems Studies (SHSS), Tata Institute of Social Sciences, V. N. Purav Marg, Deonar, 400088 Mumbai India

**Correction: Reproductive Health (2023) 20:123** **https://doi.org/10.1186/s12978-023-01667-z**

After publication of this article [1], the authors reported that in the title of this article ‘National Family Household Survey’ should have been 'National Family Health Survey’.

The original article [1] has been corrected.
