# Supplementary material for: Understanding the healthcare provider role on post abortion contraception adoption in India using National Family Health Survey-5
Source: Reprod Health. 2023 Aug 23;20:123. doi: 10.1186/s12978-023-01667-z (PMC10463293; doi:10.1186/s12978-023-01667-z)
Supplement: Supplementary file 1 — Additional file 1: S1. Percentage distribution of contraception use, permanent, spacing, and traditional method among currently married women according to status of abortion in last five years in India, NFHS-5, 2019-21. [file 12978_2023_1667_MOESM1_ESM.docx]

**S1: Percentage distribution of contraception use, permanent, spacing, and traditional method among currently married women according to status of abortion in last five years, NFHS-5, 2019-21**
