# Supplementary material for: Understanding the healthcare provider role on post abortion contraception adoption in India using National Family Health Survey-5
Source: Reprod Health. 2023 Aug 23;20:123. doi: 10.1186/s12978-023-01667-z (PMC10463293; doi:10.1186/s12978-023-01667-z)
Supplement: Supplementary file 2 — Additional file 2: S2. Percentage distribution of time to adopt a contraception method, permanent, spacing, and traditional method in last five years by type of contraception among women who undergo abortion in India, NFHS-5, 2019-21. [file 12978_2023_1667_MOESM2_ESM.docx]

**S2: Percentage distribution of time to adopt a contraception method, permanent, spacing, and traditional method in last five years by type of contraception among women who undergo abortion, NFHS-5, 2019-21**
