# Supplementary material for: Understanding the healthcare provider role on post abortion contraception adoption in India using National Family Health Survey-5
Source: Reprod Health. 2023 Aug 23;20:123. doi: 10.1186/s12978-023-01667-z (PMC10463293; doi:10.1186/s12978-023-01667-z)
Supplement: Supplementary file 3 — Additional file 3: Table S1. Adjusted Logistic regression for adoption of female sterilization post abortion in last five years by selected covariates in India, NFHS, 2019-21. [file 12978_2023_1667_MOESM3_ESM.docx]

**Table S1.** Adjusted Logistic regression for adoption of female sterilization post abortion in last five years by selected covariates, NFHS, 2019-21

|  | **AOR (95% CI)** |
| --- | --- |
| **Place of Residence** | 1.00 |
| Urban | 0.82 (0.49  1.36) |
| Rural |  |
| **Region** | 1.00 |
| Northern | 1.59 (0.67  3.78) |
| Central | 1.73 (0.76  3.94) |
| Eastern | 0.28(0.07  1.13) |
| North-Eastern | 1.84 (0.72  4.69) |
| Western | 0.66 (0.30  1.46) |
| Southern |  |
| **Caste** | 1.00 |
| SC/ST | 0.55***(0.34  0.88) |
| OBC | 0.68 (0.38  1.22) |
| Others |  |
| **Religion** | 1.00 |
| Hindu | 0.59 (0.28  1.21) |
| Non-Hindu |  |
| **Age of Women** | 1.00 |
| 15-29 | 0.99 (0.51  1.9) |
| 30-39 | 3.69 (0.63  21.49) |
| 40+ |  |
| **Educational Status** | 1.00 |
| No Education | 0.99 (0.45  2.2) |
| Primary | 0.73 (0.39  1.37) |
| Secondary | 0.25***(0.11  0.59) |
| Higher |  |
| **Child Sex Composition** |  |
| Only Son | 1.00 |
| Only Daughter | 1.19 (0.57  2.52) |
| Both | 1.21 (0.73  1.98) |
| **Parity of Women** |  |
| Less Than 2 | 1.00 |
| More than 2 | 0.16(0.02  1.12) |
| **Ideal Number of Children in Relation to Actual Living Children** |  |
| Ideal=Actual | 1.00 |
| Ideal is More than Actual | 0.79 (0.29  2.17) |
| Ideal is Less than Actual | 0.37***(0.22  0.62) |
| **Age at Abortion** |  |
| Less than 20 | 1.00 |
| 20-24 | 0.94 (0.24  3.73) |
| 25-29 | 4.03***(1.01  16.06) |
| 30+ | 6.61***(1.51  28.83) |
| **Timing of Abortion** |  |
| First Trimester | 1.00 |
| Second Trimester | 0.49***(0.28  0.86) |
| **Place of Abortion** |  |
| Home | 1.00 |
| Public | 2.01 (0.74  5.47) |
| Private | 1.65 (0.64  4.27) |
| **Person Performed Abortion** |  |
| Others/Self | 1.00 |
| Health Professional | 0.89 (0.37  2.10) |
| **Procedure Used for Abortion** |  |
| Surgical | 1.00 |
| Non-Surgical | 0.71 (0.44  1.15) |
| **Reason for Abortion** |  |
| Medical Issues | 1.00 |
| Unplanned Pregnancy | 2.15***(1.34  3.45) |
| Others | 1.47 (0.79  2.72) |
| **Compensation received for sterilization** |  |
| No | 1.00 |
| Yes | 1.70***(1.10  2.62) |
| ****p<0.05, ***p<0.00** |  |
